# Effects of NMES Combined with Resistance Training Using Underwater Surface EMG Sensors on Neuromuscular Activation of Breaststroke Technique in Breaststroke Athletes: Analysis of Non-Negative Matrix Muscle Synergy

**DOI:** 10.3390/s26020671

**Published:** 2026-01-20

**Authors:** Yaohao Guo, Tingyan Gao, Bin Kong

**Affiliations:** 1 School of Mathematical Sciences, Fudan University, Shanghai 200433, China; guoyaohao_fdu@163.com; 2 Faculty of Physical Education, Fudan University, Shanghai 200433, China; gaotingyan@fudan.edu.cn

**Keywords:** neuromuscular electrical stimulation, resistance training, breaststroke, surface electromyography, muscle synergy analysis, non-negative matrix factorization, underwater sensor

## Abstract

Background: Neuromuscular electrical stimulation (NMES) is an effective exogenous neuromuscular activation method widely used in sports training and rehabilitation. However, existing research primarily focuses on land-based sports or single-joint movements, with limited in-depth exploration of its intervention effects and underlying neuromuscular control mechanisms for complex, multi-joint coordinated aquatic activities like breaststroke swimming. This study aimed to investigate the effects of NMES combined with traditional resistance training on neuromuscular function during sport-specific technical movements in breaststroke athletes. Methods: A randomized controlled trial was conducted with 30 national-level or above breaststroke athletes assigned to either an experimental group (NMES combined with traditional squat resistance training) or a control group (traditional squat resistance training only) for an 8-week intervention. A specialized fully waterproof wireless electromyography (EMG) sensor system (Mini Wave Infinity Waterproof) was used to synchronously collect surface EMG signals from 10 lower limb and trunk muscles during actual swimming, combined with high-speed video for movement phase segmentation. Changes in lower limb explosive power were assessed using a force plate. Non-negative matrix factorization (NMF) muscle synergy analysis was employed to compare changes in muscle activation levels (iEMG, RMS) and synergy patterns (spatial structure, temporal activation coefficients) across different phases of the breaststroke kick before and after the intervention. Results: Compared to the control group, the experimental group demonstrated significantly greater improvements in single-leg jump height (Δ = 0.06 m vs. 0.03 m) and double-leg jump height (Δ = 0.07 m vs. 0.03 m). Time-domain EMG analysis revealed that the experimental group showed more significant increases in iEMG values for the adductor longus, adductor magnus, and gastrocnemius lateralis during the leg-retraction and leg-flipping phases (*p* < 0.05). During the pedal-clamp phase, the experimental group exhibited significantly reduced activation of the tibialis anterior alongside enhanced activation of the gastrocnemius. Muscle synergy analysis indicated that post-intervention, the experimental group showed a significant increase in the weighting of the vastus medialis and biceps femoris within synergy module 4 (SYN4, related to propulsion and posture) (*p* < 0.05), a significant increase in rectus abdominis weighting within synergy module 3 (SYN3, *p* = 0.033), and a significant shortening of the activation duration of synergy module 2 (SYN2, *p* = 0.007). Conclusions: NMES combined with traditional resistance training significantly enhances land-based explosive power in breaststroke athletes and specifically optimizes neuromuscular control strategies during the underwater breaststroke kick. This optimization is characterized by improved activation efficiency of key muscle groups, more economical coordination of antagonist muscles, and adaptive remodeling of inter-muscle synergy patterns in specific movement phases. This study provides novel evidence supporting the application of NMES in swimming-specific strength training, spanning from macroscopic performance to microscopic neural control.

## 1. Introduction

Neuromuscular Electrical Stimulation (NMES) has gained widespread application in athletic training and rehabilitation as an effective exogenous neuromuscular activation method [1]. Its principle involves applying electrical currents through surface electrodes to directly or reflexively excite motor nerves, inducing contraction in target muscles to enhance muscle strength and improve neuromuscular control [2]. In competitive sports, integrating NMES with traditional resistance training aims to achieve deeper stimulation and remodeling of the athlete’s neuromuscular system through the synergistic effects of “voluntary contraction” and “electrically induced contraction”. This approach has become a cutting-edge research direction in strength training [3]. Research indicates that NMES-augmented training effectively enhances muscle power, maximum strength, and muscle activation efficiency in both healthy athletes and patients [4]. However, existing studies predominantly focus on land-based sports or single-joint movements. In-depth exploration remains lacking regarding the intervention effects and underlying neuromuscular control mechanisms for complex, multi-joint coordinated aquatic activities like swimming.

As a primary swimming technique, the breaststroke relies heavily on the cyclical “grip–flip–kick–squeeze” leg action for propulsion. This movement demands highly coordinated explosive contraction and precise control from lower-body muscle groups while overcoming substantial fluid resistance [5]. Consequently, the activation patterns, coordination capabilities, and force output efficiency of the lower-limb neuromuscular system directly determine a breaststroke swimmer’s technical performance and athletic outcomes [6]. While traditional resistance training enhances absolute muscle strength, its specific transfer effects on the neural drive patterns of aquatic-specific technical movements remain limited [7]. Investigating whether NMES combined with resistance training can optimize neuromuscular function during specialized technical movements in breaststroke athletes holds significant value for enriching specialized training theories and methodologies.

Surface Electromyography (sEMG) signals serve as the gold standard for examining neuromuscular activation states. Time-domain analysis of sEMG signals quantifies muscle activation levels and recruitment patterns [8]. However, time-domain metrics struggle to reveal the synergistic strategies employed by multiple muscles during complex tasks. In recent years, muscle synergy analysis—particularly methods based on non-negative matrix factorization (NMF)—has emerged as a powerful tool for understanding the modular organization principles governing the central nervous system’s (CNS) control of complex movements [9]. This approach decomposes multi-channel EMG signals into a small number of fixed spatial synergistic patterns (reflecting weighted muscle combinations) and their corresponding temporal activation coefficients, thereby reducing the complexity of multidimensional control to the allocation of several synergistic modules [9]. This approach decomposes multi-channel EMG signals into a small number of fixed spatial synergy patterns (reflecting weighted muscle combinations) and their corresponding temporal activation coefficients, thereby reducing complex multidimensional control to the allocation of several synergistic modules. This provides a more profound perspective than traditional time-domain metrics for quantitatively assessing the impact of training interventions on neuromuscular control “patterns”.

In the field of movement science, innovations in sensor technology continue to advance research. Notably, the development of wearable and wireless sensing systems enables real-time collection of physiological and biomechanical data during dynamic, real-world movement scenarios. Inertial measurement units (IMUs) and flexible pressure sensors are already widely applied in land-based sports for technical/tactical analysis and injury risk assessment [10]. However, aquatic environments present unique challenges—including water conductivity, pressure, and signal transmission—resulting in a relative scarcity of sensor technologies capable of stable, reliable acquisition of high-quality physiological signals (particularly sEMG) [11]. Most underwater electromyography studies are constrained by equipment limitations, often conducted under static or simulated conditions, making it difficult to fully reflect muscle activity during actual swimming dynamics. Therefore, developing and applying specialized underwater electromyography sensing systems is a key prerequisite for studying swimming neuromuscular mechanisms in environments with higher ecological validity. It also represents a critical technical challenge and research innovation point in this field that urgently requires breakthroughs.

In particular, the few existing swimming-related studies are constrained by technological limitations, often relying on land-based simulations or static underwater measurements. These approaches lack the ecological validity to systematically explore neuromuscular control mechanisms during real, dynamic aquatic movements, especially for complex, multi-joint coordination tasks such as the breaststroke kick. Consequently, there remains a significant gap in understanding how NMES interventions modulate sport-specific neuromuscular strategies in authentic swimming contexts.

Against this backdrop, this study introduces a specialized fully waterproof wireless EMG sensor system (Mini Wave Infinity Waterproof) to overcome the underwater limitations of traditional equipment. This enables high-quality, synchronous acquisition of multi-muscle EMG signals during actual swimming by breaststroke athletes. This study employs a randomized controlled trial to investigate the effects of an 8-week NMES program combined with traditional resistance training (using squats as the exercise modality) on the neuromuscular function of national-level and above breaststroke athletes. The study will not only evaluate changes in lower-body explosive power using a force plate but will also focus on combining NMF muscle coordination analysis methods to deeply analyze alterations in activation levels (iEMG, RMS) and coordination patterns (spatial structure, temporal activation coefficients) of lower-body and trunk muscles during specialized breaststroke kicking movements before and after the intervention. Accordingly, this study hypothesizes that (1) NMES-combined training will lead to greater improvements in lower-body explosive power compared to traditional resistance training alone; (2) it will enhance the activation efficiency of key propulsive muscles during critical phases of the breaststroke kick (e.g., leg-retraction and pedal-clamp phases); and (3) it will induce specific remodeling of muscle synergy patterns, characterized by increased weighting of primary movers and more economical temporal activation profiles.

## 2. Participants and Methods

### 2.1. Participants

This study employed G*Power 3.1 software for a pre-test sample size calculation. Based on an expected effect size (Cohen’s *d* = 0.80), an α level of 0.05, a statistical power (1 − β) of 0.80, and using repeated measures ANOVA (between-group × time interaction), the results indicated a minimum sample size of 12 participants per group. Accounting for potential attrition (approximately 20%), the final sample size was set at 30 participants (15 per group) to ensure sufficient statistical power for detecting significant effects of the training intervention. This study included 30 breaststroke athletes. All subjects held national first-class athlete status or higher, had competed in national championships and other national-level events, and achieved top-three finishes. The experimental group athletes had an average professional training duration of 8.5 ± 2.1 years. Their average age was 18.6 ± 1.8 years, average height 182.1 ± 5.2 cm, and average weight 68.3 ± 4.7 kg. The control group athletes had an average professional training duration of 8.7 ± 2.0 years. Their average age was 18.9 ± 1.6 years, average height was 180.8 ± 5.5 cm, and average weight was 67.9 ± 5.0 kg. Basic information on participants is shown in Table 1. This research protocol has been approved by the Ethics Review Committee. All participants were fully informed of the study’s purpose, procedures, potential risks, and benefits prior to the experiment and voluntarily signed written informed consent forms. The research process strictly adhered to the principles of the Declaration of Helsinki, safeguarding participants’ privacy rights and freedom to withdraw.

Inclusion Criteria:

① Fully understand the study content and potential risks, voluntarily participate, and sign an informed consent form;

② Meet the basic requirements for the recruitment group;

③ No significant physiological or psychological disorders;

④ No lower limb skeletal or muscular surgical procedures within the past year.

Exclusion Criteria:

① Presence of the aforementioned disorders or injuries;

② Refusal to participate in testing or failure to sign the informed consent form;

③ History of prolonged absence from systematic professional training.

### 2.2. Research Methods

#### 2.2.1. Experimental Equipment

(1) Mini Wave Infinity Waterproof:

Mini Wave Infinity Waterproof is a CE-certified professional underwater electromyographic biofeedback and electrical stimulation system (CHATTANOOGA, Austin, TX, USA). Its core modules include neuromuscular re-education, aquatic strength training, pain management, and rehabilitation. Featuring a fully waterproof design, the system enables simultaneous electromyographic signal monitoring and electrical stimulation therapy (frequency: 2000 Hz) underwater, delivering real-time biofeedback.

(2) EMG and Motion Tools:

EMG and Motion Tools (5.2) is the professional analysis software bundled with the Mini Wave Infinity Waterproof System. It synchronously processes underwater electromyography (EMG) and motion data—such as integrated EMG (iEMG) and muscle activation timing—enabling precise quantitative assessment.

(3) GoPro HERO12 Black:

The GoPro HERO12 Black supports 4 K/120 fps and 1080 p/240 fps high-speed video recording, enabling clear capture and significant slow-motion playback to highlight detailed motion breakdowns (GoPro Inc., San Mateo, CA, USA).

(4) Kinovea:

Kinovea (Joan Charmant (Project Lead), France) is a specialized video analysis tool designed for analyzing kinematic parameters of synchronously captured motion videos. It enables precise segmentation of movement phases. Through frame-by-frame playback, it can decompose continuous muscle exertion processes (such as a complete kick or stroke) into multiple specific action phases based on predetermined criteria, while quantifying the duration and sequence of each phase.

(5) Kistler 3D Force Plate

The Kistler 3D force plate (Model 9286A, dimensions 90 cm × 60 cm × 10 cm, sampling frequency 1000 Hz, Kistler Group, Winterthur, Switzerland) precisely measures forces, torques, and pressure centers along the X, Y, and Z axes for biomechanical research and industrial applications via piezoelectric sensors. It was employed in this study for ground reaction force data acquisition.

(6) My Jump 2 Software

The Kistler 3D force platform features an A15 bionic chip and a 5-core graphics processor, equipped with a 12-megapixel main camera and a 12-megapixel ultra-wide-angle dual-camera system. The force platform comes with built-in My Jump 2 software for collecting and analyzing jump data. This software records the jump process in slow-motion mode via high-speed cameras, automatically calculating key parameters such as vertical jump height, velocity, force, and power. Experiments should be conducted in an indoor environment with uniform lighting and a level surface.

(7) Other Equipment: Electrode pads, waterproof EMG washers, waterproof skin film, scissors, adhesive tape, rubbing alcohol, cotton balls, stopwatch, razor.

#### 2.2.2. Intervention Protocol

This study employed a randomized crossover design. The experimental group received NMES combined with traditional resistance training intervention, while the control group received only traditional resistance training intervention.

The NMES electrodes were positioned in strict accordance with SENIAM guidelines for surface electromyography placement, targeting motor points of the quadriceps femoris. Specifically, for the large-area electrodes (10 cm × 5 cm), the proximal edge was aligned 5 cm superior to the superior patellar border. For the standard electrodes (5 cm × 5 cm) placed longitudinally along the rectus femoris belly, the center was positioned at 50% of the distance between the anterior superior iliac spine and the superior patellar border. The electrical stimulation parameters (25–35 mA, 300 μs, 10–18 Hz during squat descent; 10 mA, 3 Hz during relaxation) were selected based on a combination of established NMES protocols for strength training and individual tolerance adjustments during preliminary familiarization sessions, aiming to evoke strong yet comfortable contractions without causing fatigue or discomfort.

##### Intervention Protocol for the Experimental Group

1. Subjects in the experimental group must complete NMES combined with traditional resistance training, training three times per week for eight weeks. NMES intervention will be synchronized with each resistance training session in a fixed sequence.
2. Electrodes were applied using the Mini Wave Infinity Waterproof System’s dedicated waterproof electrodes. One pair of large-area electrodes (10 cm × 5 cm) was placed proximally on both quadriceps femoris muscles. Two pairs of standard electrodes (5 cm × 5 cm) were applied longitudinally along the rectus femoris muscle belly, with specific placement guided by the system’s included muscle trigger point map. All electrodes must be rigorously sealed with waterproof gaskets and waterproof skin membranes to ensure signal quality underwater.
3. Prior to formal training, a 5 min standardized warm-up with submaximal electrical stimulation intensity is performed. Subsequently, during the athlete’s barbell squat execution, a fixed-intensity pulsed stimulation is continuously applied from the start of the descent until just before standing. Upon standing, the stimulation transitions to a low-intensity relaxation phase. Stimulation duration per squat cycle is 4 s, with pulse intensity set at 25–35 mA, pulse width 300 μs, and frequency 10–18 Hz. The relaxation phase lasts 2 s at 10 mA intensity and 3 Hz frequency. Barbell squat load is set at 65% of 1 RM. Each complete training session comprises 4 sets, with 20 cycles performed per set. Rest periods between sets are 30 s.

##### Control Group Intervention Protocol

Control group participants underwent only traditional resistance training (the same squat training as the experimental group), using a load of 65% 1 RM. They completed 4 sets of 20 repetitions each, with 30 s of rest between sets (Table 2).

### 2.3. Data Acquisition

Baseline data collection, including patient demographics and electromyography measurements, was conducted the day before the intervention began. Post-intervention data collection took place the day after the intervention concluded.

#### 2.3.1. Explosive Power Data

The test utilizes a force plate, with the following movements: single-leg jump landing and double-leg jump landing.

First, perform the single-leg jump landing test: The subject stands with both feet on the force plate. Upon hearing the “Go” command, jump upward as high as possible, then land on one foot and maintain stability for 3 s.

After a 3 min rest period, the two-foot landing test is performed: The subject stands with both feet on the force plate. Upon hearing the “Go” command, they jump upward as high as possible, then land with both feet and maintain stability for 3 s.

#### 2.3.2. Surface Electromyography Data

Using the Mini Wave infinity Waterproof EMG System (sampling frequency 2000 Hz) for aquatic testing, synchronized with a GoPro HERO12 Black high-speed camera, to jointly collect electromyographic and kinematic data during the breaststroke motion. Target muscles included: right lower limb muscle groups (Tibialis anterior, Gastrocnemius medialis, Gastrocnemius lateralis, Adductor longus, Adductor magnus, Biceps femoris, Gluteus maximus) and right trunk muscle groups (Rectus abdominis, Latissimus dorsi, Trapezius), totaling 10 muscles. Prior to testing, subjects underwent skin preparation: surface hair removal, 75% alcohol skin cleansing, electrode placement following SENIAM guidelines, and rigorous sealing with waterproof gaskets and waterproof skin membranes [12].

**Figure 1:**
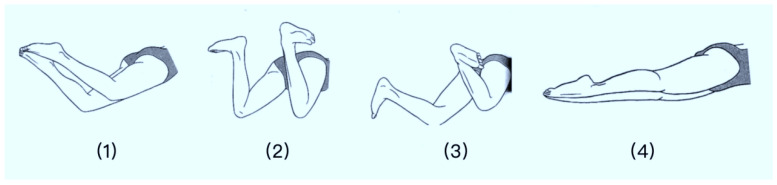
Phases of the Breaststroke Leg Kick.

Based on the biomechanical characteristics of the breaststroke, a complete breaststroke cycle was divided into four consecutive phases: leg recovery, foot rotation, kick-squeeze, and glide [13], as shown in Figure 1:

(1) Leg Retraction Phase: From the initiation of leg retraction to the kick-and-squeeze moment;
(2) Leg Rotation Phase: From the moment of active ankle outward and backward rotation to the kick-and-squeeze moment;
(3) Kick-and-Squeeze Phase: From the kick-and-squeeze moment to the glide phase;
(4) Glide Phase: From the onset of gliding to the conclusion of the glide.

Electromyographic signals from designated muscles were recorded during the breaststroke movement. Non-negative matrix factorization (NMF) was then applied to extract muscle coordination features, investigating the effects of different interventions on muscle coordination patterns.

### 2.4. Data Processing

#### 2.4.1. Data Extraction and Preprocessing

During each test session, participants performed three consecutive 25 m breaststroke trials at race pace, from which a minimum of five complete stroke cycles (from leg recovery to glide) were extracted for analysis. The middle three cycles from each trial were selected to minimize start and turn effects, and EMG signals from corresponding phases were ensemble-averaged to reduce within-subject variability. Synchronization between the Mini Wave Infinity EMG system and the GoPro HERO12 Black camera was achieved via an external digital trigger signal sent simultaneously to both devices at the start of each trial, ensuring precise temporal alignment. Video data were analyzed using Kinovea software (2025.1) to segment each cycle into four phases (leg-retraction, leg-flipping, pedal-clamp, glide), with phase boundaries defined by key kinematic events (e.g., maximal knee flexion, ankle rotation onset).

1. Using data synchronously acquired from a surface electromyography (sEMG) system with a high-speed camera and 2000 Hz sampling frequency, Kinovea video analysis software was employed to delineate the complete cycle of each breaststroke movement (from the pull-through to the glide phase). High-frequency sEMG data corresponding to this segment were extracted based on synchronized timing signals for subsequent analysis.
2. Batch preprocessing of raw surface electromyography (sEMG) signals was performed in MATLAB (R2019b) [14]. The processing workflow included (1) applying a 10–400 Hz bandpass filter to remove noise; (2) removing signal mean to eliminate DC offset; (3) performing full-wave rectification on the signal; (4) obtaining the linear envelope using a 4th-order zero-phase shift Butterworth low-pass filter with a cutoff frequency of 10 Hz; (5) normalizing the EMG signal amplitude to the peak activation value of its respective channel during a specific action to eliminate differences in activation levels between muscles; (6) time-normalizing the EMG signal for each phase to 101 data points.

#### 2.4.2. Electromyographic Time-Domain Analysis

Time-domain analysis of preprocessed electromyographic signals primarily extracts integral electromyography (iEMG) and root mean square (RMS) amplitude [15]. iEMG is obtained by time-integrating the full-wave rectified electromyographic signal. Its value reflects the total amount of muscle electrical activity over a specific time period, serving as a comprehensive indicator for assessing the overall mobilization level of the neuromuscular system. The formula is as follows:

x_n_ represents the amplitude value of the electromyographic signal at the nth sampling point (typically measured in microvolts, μV). This signal has undergone full-wave rectification, so taking the absolute value ensures all values are positive. |x_n_| denotes the absolute value of the electromyographic signal at each sampling point. N denotes the total number of sampling points within the selected time window. Σ (from n = 1 to N) represents the cumulative sum of the absolute values across all sampling points. IEMG is the result of this accumulation (typically measured in μV·s), reflecting the total amount of muscle electrical activity during the analyzed time period. A higher IEMG value indicates greater muscle activation intensity or longer activation duration. This is a summation.

$$IEMG=\sum_{n=1}^{N}\left|x_{n}\right|$$

#### 2.4.3. Muscle Synergy Analysis

##### Synergy Extraction and Quantification

Muscle synergies were extracted from preprocessed electromyographic envelope data using the non-negative matrix factorization (NNMF) algorithm [16]. NNMF decomposes the muscle activation pattern matrix V (number of muscles × time points) into the product of spatial components W (number of muscles × number of synergies) and corresponding temporal coefficients H (number of synergies × time points), as follows:

V is the raw electromyography signal matrix, dimension m × n: m denotes the number of muscle channels (10 in this study), n denotes the number of time sampling points.

W is the spatial synergy matrix, dimension m × k: each column represents a synergy module, containing the weights of each muscle within that module, k is the number of synergies extracted.

H is the temporal activation coefficient matrix, dimension k × n: each row describes the activation timing of the corresponding synergistic module during the action cycle.

e is the residual matrix, representing the signal portion not explained by the model.

This model decomposes multi-channel EMG signals into a linear combination of a small number of synergistic modules through matrix multiplication (W × H), thereby revealing the central nervous system’s control strategy for multi-muscle coordination.

$$V_{m\times n}=W_{m\times k}\times H_{k\times n}+e$$

The number of synergies k is determined by calculating the variance-explained fraction (VAF). The minimum k value where VAF first exceeds 0.9 is selected as the number of synergies for the individual. Subsequently, the spatial component W is normalized such that the maximum weight of any muscle within a synergy equals 1. If a muscle’s average weight within a synergy ≥ 0.3, it is considered a major contributor to that synergy, as per the following formula:

SSE (Sum of Squared Errors) represents the total sum of errors between the reconstructed signal from the synergistic model and the original signal;

SST (Sum of Squares Total) represents the total variance of the original electromyographic signal.

This metric reflects the proportion of variation in the original EMG signal that can be explained by the extracted synergistic modules. A VAF value closer to 1 indicates better model reconstruction performance. In research, a threshold of VAF > 0.90 is typically used to determine the number of synergies, ensuring the preservation of primary motor control patterns while avoiding overfitting.

$$\mathrm{VAF}=1-\frac{\mathrm{SSE}}{\mathrm{SST}}$$

##### k-Means Clustering Analysis

To reveal unique synergistic patterns before and after intervention, we applied k-means clustering to four datasets (pre/post-test for the experimental group and control group) to obtain reference synergistic structures for each group. The number of clusters k was determined using the inflection point method and SSE trend analysis: as k increased to s, SSE was minimally affected by random initialization, and the number of matched synergies increased. When k = s + 1, SSE showed no significant decline, and thus k = s was adopted. Taking the pre-test of the control group as an example, s reference synergies (SYN1~SYNs) were obtained. The synergy structures W of each subject within the group were matched with the reference synergies using Pearson correlation (r > 0.6 was considered similar). After matching, the corresponding temporal coefficients H were automatically grouped into the same category [17].

### 2.5. Statistical Analysis

All experimental data were statistically processed and analyzed using Excel and SPSS 26.0. Descriptive statistics in this study are presented as mean ± standard deviation (X^−^ ± SD). Outliers were removed using box plots, and data normality was verified via Shapiro–Wilk tests. For normally distributed data, a 2 × 2 (group, time) repeated-measures ANOVA was performed to analyze intergroup differences in IEMG, RMS, number of synergistic muscles, muscle weighting, activation duration, peak activation time, and onset time before and after intervention. Post hoc multiple comparisons were performed using Bonferroni-corrected paired *t*-tests. For non-normally distributed data, Friedman’s test was applied, followed by Holm-corrected Wilcoxon signed-rank tests for multiple comparisons, with effect size r reported. Results are presented as mean ± standard deviation, with significance set at *p* < 0.05 [18]. In the repeated-measures ANOVA, when a significant interaction effect (time × group) was detected, simple effects analyses were conducted with Bonferroni correction applied to adjust for multiple comparisons. For muscle synergy extraction via non-negative matrix factorization (NMF), the number of synergies (k) for each subject was determined as the smallest integer for which the variance accounted for (VAF) first exceeded 0.90, a threshold commonly adopted in motor control literature to balance between model simplicity and reconstruction accuracy. This criterion ensures that the extracted synergies capture the predominant activation patterns while minimizing overfitting to noise or individual variability.

## 3. Results

### 3.1. Effects of NMES Combined with Traditional Resistance Training on Explosive Power in Breaststroke Athletes

The research findings are presented in Table 3. NMES combined with traditional resistance training significantly increased leg explosive power in breaststroke swimmers. In the single-leg landing jump task, repeated measures ANOVA revealed a significant interaction effect between time and group (*p* = 0.016). Subsequent simple effects analysis revealed no significant difference in jump height between the experimental and control groups at baseline (*p* = 0.782). At post-intervention, the experimental group exhibited significantly greater jump height than the control group (*p* = 0.021). Furthermore, within the experimental group, the post-test jump height was significantly higher than the pre-test (*p* < 0.001). Within the control group, the post-test jump height was also significantly higher than the pre-test (*p* = 0.042), but the magnitude of improvement (Δ = 0.06 m) was greater in the experimental group than in the control group (Δ = 0.03 m).

For the double-foot landing jump, repeated measures ANOVA revealed a significant interaction effect of time × group (*p* = 0.004). Simple effects analysis indicated no significant difference in jump height between the experimental and control groups at baseline (*p* = 0.934). At post-intervention, the experimental group exhibited significantly higher jump height than the control group (*p* = 0.033). Furthermore, within the experimental group, the post-test jump height was significantly higher than the pre-test (*p* < 0.001). Within the control group, the post-test jump height was also significantly higher than the pre-test (*p* = 0.044), but the magnitude of improvement in the experimental group (Δ = 0.07 m) was greater than that in the control group (Δ = 0.03 m).

### 3.2. Effects of NMES Combined with Traditional Resistance Training on Muscle Activation During Breaststroke in Swimmers

#### 3.2.1. Integral Electromyography Values

During the breaststroke kick phase, as shown in Table 4, significant time × group interaction effects were observed for iEMG data in the gastrocnemius (*p* = 0.031), vastus medialis (*p* = 0.016), vastus lateralis (*p* = 0.004), and biceps femoris (*p* = 0.039), and gluteus maximus (*p* = 0.029) exhibited significant time × group interaction effects in iEMG data. Simple effects analysis revealed no significant differences in iEMG between the experimental and control groups for the gastrocnemius, vastus medialis, vastus lateralis, biceps femoris, and gluteus maximus muscles at baseline (*p* > 0.05). At post-test, iEMG in the gastrocnemius, vastus medialis, and vastus lateralis muscles of the experimental group was significantly higher than that of the control group (*p* < 0.05). Additionally, within both the experimental and control groups, post-test iEMG for all five muscles was significantly higher than pre-test levels (*p* < 0.05). Significant time × group interaction effects were observed for iEMG data in the vastus lateralis (*p* = 0.018) and rectus abdominis (*p* = 0.042) during the leg-flipping phase. Simple effects analysis revealed no significant differences in iEMG between the experimental and control groups for either muscle during the pre-test (*p* > 0.05). During the post-test, the iEMG of the Rectus abdominis in the experimental group was significantly higher than that in the control group (*p* < 0.05). Furthermore, within both the experimental and control groups, post-test iEMG values for both muscles were significantly higher than pre-test values (*p* < 0.05). Significant time × group interaction effects were observed for iEMG data in the Tibialis anterior (*p* = 0.048) and Gastrocnemius (*p* = 0.042) during the pedal-clamp phase. Simple effects analysis revealed no significant differences in iEMG between the experimental and control groups for either muscle during the pre-test (*p* > 0.05). During the post-test, the iEMG of the Tibialis anterior in the experimental group was significantly lower than that in the control group (*p* < 0.05). Furthermore, within the experimental group, significant differences existed in iEMG for both muscles between pre- and post-test (*p* < 0.05). Within the control group, iEMG for the gastrocnemius muscle at post-test was significantly higher than at pre-test (*p* < 0.05). No significant differences in iEMG were observed for any of the ten muscles during the gliding phase.

#### 3.2.2. Effects of NMES Combined with Traditional Resistance Training on Muscle Synergy in the Breaststroke Movement of Swimmers

##### Muscle Synergy Patterns in the Breaststroke Movement

**Figure 2:**
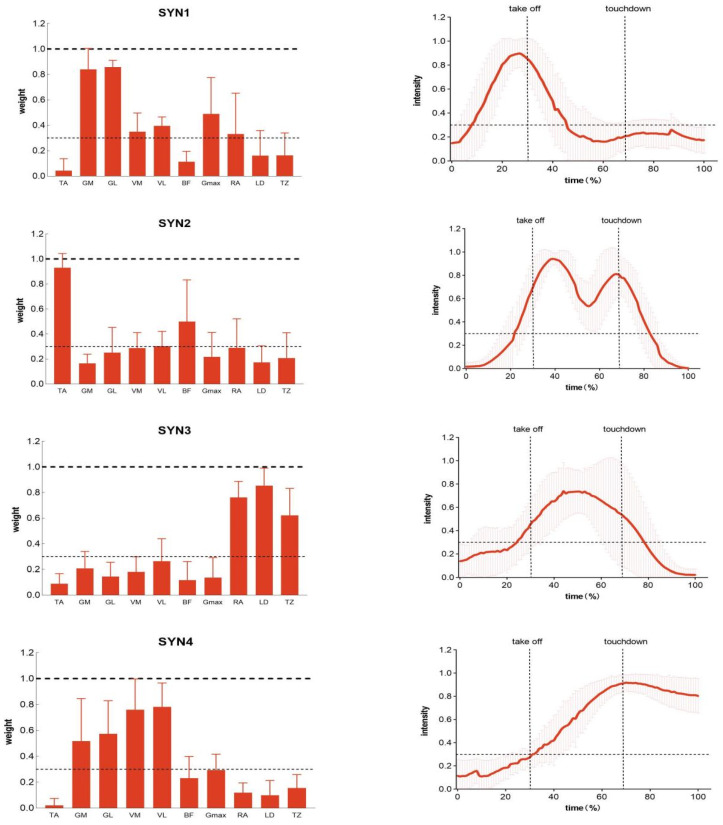
Schematic diagram illustrating the extraction of synergistic movements in the breaststroke action (data presented represents the mean values of the control group prior to intervention). TA: Tibialis anterior; GM: Gastrocnemius medialis; GL: Gastrocnemius lateralis; VM: Adductor longus; VL: Adductor magnus; BF: Biceps femoris; Gmax: Gluteus maximus; RA: Rectus abdominis; LD: latissimus dorsi; TZ: trapezius. Data are presented as mean values; error bars represent ±1 standard deviation (SD).

In the breaststroke movement, pre-intervention electromyography data from the control group clustered into four reference synergies. Figure 2 illustrates the muscle synergy patterns matched to these four reference synergies under pre-intervention conditions in the control group. Referencing breaststroke motion analysis literature and activation coefficients extracted from synergies, we analyzed the primary functions performed by the four synergies. Synergy 1 primarily functions during the Leg-retraction phase; Synergy 2 exhibits activation peaks during the Leg-flipping phase; Synergy 3 activates mainly during the Kick and Leg-squeeze phases; Synergy 4 functions during the Gliding phase following the Kick-extension.

##### Synergistic Structure in the Breaststroke Movement

As shown in Table 5, in Synergy I, the primary activated muscles were the medial and lateral gastrocnemius. Moderate activation was also observed in the medial and lateral vastus muscles, gluteus maximus, and rectus abdominis. Repeated measures ANOVA revealed that within the experimental group, gluteus maximus weight significantly decreased post-intervention (*p* = 0.043). Within the control group, gluteus maximus weight showed an upward trend post-intervention but no significant difference existed between pre- and post-intervention. Furthermore, group differences did not significantly alter muscle activation weights either pre- or post-intervention.

As shown in Table 6, in Synergy II, the dominant muscle is the Tibialis anterior, with moderate activation also observed in the Biceps femoris. Comparisons using repeated measures ANOVA revealed no significant differences in the synergy weights of the ten muscles between the two groups before and after intervention.

As shown in Table 7, the primary activated muscles during Synergy III were the rectus abdominis, latissimus dorsi, and trapezius. Repeated measures ANOVA revealed that within the experimental group, the weighting of the Rectus abdominis significantly increased post-intervention (*p* = 0.033). Within the control group, no significant difference in Rectus abdominis weighting was observed before and after intervention. Furthermore, intergroup differences did not significantly alter muscle activation weighting at either pre- or post-intervention time points.

As shown in Table 8, during Synergy IV, the primary activated muscles were the vastus medialis and laterals, as well as the gastrocnemius medialis and lateralis. The biceps femoris and gluteus maximus also exhibited a certain degree of activation. Comparisons using repeated measures ANOVA revealed significant interaction effects for the activation weights of the vastus medialis (*p* = 0.007) and biceps femoris (*p* = 0.014). Within the experimental group, activation weights for the vastus medialis and biceps femoris significantly increased post-intervention. Within the control group, no significant differences in activation weights for the vastus medialis and biceps femoris were observed before and after intervention. Furthermore, group differences did not significantly alter muscle activation weights either before or after intervention.

##### Activation Coefficient in Breaststroke Movements

As shown in Table 9, during the coordination of the breaststroke movement, the coordination curve begins activation in the leg-retraction phase, rapidly rises to peak, and then rapidly declines. The activation phase is primarily concentrated during the leg-retraction period. There were no significant differences in peak activation timing across the four conditions, occurring around 25–30% of the stroke duration and consistently near the leg-flip moment. No significant main effects or interaction effects were observed for activation duration, peak timing, or onset timing before and after the two intervention groups. In the activation coefficient of Synergy II, the synergy curve begins activation before leg extension, persists throughout the Pedal-clamp stage, and declines after extension. Repeated measures ANOVA revealed a significant interaction effect for activation duration. Within the experimental group, activation duration significantly decreased post-intervention (*p* = 0.007); within the control group, no significant difference existed in activation duration before and after intervention. Furthermore, no significant between-group differences in activation duration were observed either pre- or post-intervention. Neither the peak timing nor the onset timing showed significant main effects or interaction effects across groups before or after intervention. For Synergy 3 activation coefficients, activation commenced before the pedal stroke, gradually increased to peak, then slowly decreased. The activation phase primarily occurred during the pedal-clamp stage. Peak activation timings across the four conditions showed no significant differences, occurring around 37–46% and consistently within the pedal-clamp stage. No significant main effects or interaction effects were observed for activation duration, peak timing, or onset timing before and after intervention in either group. For Synergy 4, the activation phase was primarily concentrated during the coasting period. There were no significant differences in peak activation timing across the four conditions, which occurred around 64–75% and were all near the coasting phase. No significant main effects or interaction effects were observed for activation duration, peak timing, or onset timing before and after intervention in either group.

## 4. Discussion

This study investigated the effects of NMES combined with resistance training on neuromuscular activation patterns and explosive power in elite breaststroke swimmers, using waterproof surface EMG sensors. The findings demonstrate that NMES-enhanced training significantly improves lower-limb explosive power on land and optimizes neuromuscular coordination during underwater breaststroke kicking, providing novel evidence linking NMES intervention to both performance outcomes and neural control strategies.

The experimental group showed greater gains in single- and double-leg jump height than the control group, consistent with existing evidence on NMES for enhancing explosive power [19]. Beyond traditional mechanisms such as increased muscle cross-sectional area, NMES may augment neural drive by directly recruiting high-threshold motor units and enhancing corticospinal excitability [20,21]. This was reflected in elevated iEMG and RMS values in key muscles such as the adductor longus, adductor magnus, and gastrocnemius lateralis during preparatory phases of the kick, suggesting improved pre-activation and energy storage for propulsion.

During the pedal-clamp phase, reduced tibialis anterior activation alongside enhanced gastrocnemius activation indicates more economical agonist–antagonist coordination. This likely stems from NMES-induced adaptations in spinal reciprocal inhibition or cortical suppression of unnecessary co-activation, promoting selective motor recruitment [22]. Such refined control may reduce internal resistance and improve propulsion efficiency, as supported by increased quadriceps activation during the glide phase for posture maintenance [23].

Muscle synergy analysis revealed specific restructuring of neuromuscular modules. In Synergy 4—associated with propulsion and glide—increased weighting of the vastus medialis and biceps femoris suggests enhanced knee stability and intermuscular coordination. The biceps femoris, as a two-joint muscle, may facilitate finer control of knee extension and hip positioning, smoothing the transition between recovery and propulsion [24,25]. Increased rectus abdominis contribution in Synergy 3 points to improved core engagement and force transmission [26]. Furthermore, shortened activation duration of Synergy 2 (tibialis anterior-dominant) aligns with the observed reduction in antagonist co-activation, reinforcing more transient and task-specific ankle control [27].

Unlike previous studies limited to land-based or simulated conditions, this research employed a waterproof EMG system to capture authentic swimming dynamics, enhancing ecological validity. Nevertheless, some limitations should be noted: the sample consisted solely of elite athletes, limiting generalizability; upper-limb muscles were not assessed; the 8-week intervention precludes conclusions on long-term effects; and the NMF method, while widely used, entails inherent constraints such as solution non-uniqueness.

Limitations and Future Research Directions: Although this study employed a rigorous randomized controlled design and advanced sensor technology to capture in-water neuromuscular activity, some methodological considerations warrant mention. First, muscle synergy analysis based on non-negative matrix factorization (NMF) can be conceptually complex and its interpretation requires familiarity with motor control theory. To enhance accessibility, future studies could incorporate more intuitive visualizations or simplified descriptors of synergy function in relation to swimming phases. Second, the synergy models were derived and validated within the same cohort of elite breaststroke athletes. While this approach is common in exploratory research, the generalizability of these specific synergy patterns to other populations (e.g., recreational swimmers, different stroke specialists) or their predictive validity in new individuals remains to be tested. Future work should include external validation using an independent sample to confirm the robustness and transferability of the identified neuromuscular modules.

## 5. Conclusions

This study demonstrates that an 8-week NMES-combined resistance training program not only improves lower-limb explosive power in elite breaststroke athletes but also optimizes sport-specific neuromuscular control during the breaststroke kick. Key adaptations include enhanced activation of primary propulsive muscles, more economical agonist–antagonist coordination, and remodeled muscle synergy patterns. These findings support the integration of NMES into swimming-specific strength training regimens to enhance both performance and movement efficiency.

## Figures and Tables

**Table 1:** Participant Characteristics (*n* = 30).

Group	Age (Years)	Height (cm)	Weight (kg)	Training Duration (Years)
Experimental group	18.6 ± 1.8	182.1 ± 5.2	68.3 ± 4.7	8.5 ± 2.1
Control group	18.9 ± 1.6	180.8 ± 5.5	67.9 ± 5.0	8.7 ± 2.0

The experimental group underwent NMES combined with resistance training, while the control group underwent traditional resistance training.

**Table 2:** Intervention Protocol.

Item	NMES Combined with Traditional Resistance Training Group	Traditional Resistance Training Group
Intervention	Squat combined with electrical stimulation	Squat
Movement Requirements	Electrical stimulation initiates when knee flexion reaches 90°	Feet positioned approximately 1.5 times shoulder-width apart, toes turned out 10–15°, squat until thighs are 90–100° relative to the ground
Load Intensity	65% 1 RM	65% 1 RM
Electrical Stimulation Parameters	Squat phase: 25–35 mA, 300 µs, 10–18 Hz Relaxation phase: 10 mA, 3 Hz	/
Sets & Repetitions	4 sets × 20 repetitions	4 sets × 20 repetitions
Rest Between Sets	30 s	30 s

**Table 3:** Effects of NMES Combined with Traditional Resistance Training on Leg Explosive Power in Breaststroke Athletes (M ± SD, m).

Test Action	Experimental Group		Control Group	
	Pre-Intervention	Post-Intervention	Pre-Intervention	Post-Intervention
One-legged jump	0.29 ± 0.09	0.35 ± 0.11 *	0.28 ± 0.07	0.31 ± 0.13
Double-foot jump	0.51 ± 0.14	0.58 ± 0.25 *	0.52 ± 0.18	0.55 ± 0.16

Note: *: Time × Group interaction effect, *p* < 0.05.

**Table 4:** Effects of NMES Combined with Traditional Resistance Training on iEMG in the Breaststroke Movement of Swimmers (M ± SD, μV·s).

Muscle	Experimental Group		Control Group	
	Pre-Intervention	Post-Intervention	Pre-Intervention	Post-Intervention
Leg-retraction phase				
Tibialis anterior	36.36 ± 8.84	39.82 ± 9.78	39.71 ± 7.08	33.12 ± 10.85
Gastrocnemius medialis	147.37 ± 17.94	155.66 ± 20.28	152.90 ± 20.14	161.84 ± 26.47
Gastrocnemius lateralis	154.47 ± 15.89	178.62 ± 20.91 *	149.74 ± 18.22	163.58 ± 24.32
Adductor longus	167.08 ± 17.84	201.87 ± 22.76 *	163.71 ± 21.86	184.24 ± 27.79
Adductor magnus	162.89 ± 18.55	213.96 ± 23.48 *	167.80 ± 23.94	191.17 ± 31.60
Biceps femoris	64.60 ± 11.67	87.12 ± 12.62 *	66.16 ± 13.71	78.42 ± 15.52
Gluteus maximus	122.86 ± 15.85	150.17 ± 19.82 *	130.73 ± 16.03	146.38 ± 20.63
Rectus abdominis	63.80 ± 11.23	66.37 ± 13.09	60.83 ± 10.79	57.53 ± 12.79
Latissimus dorsi	47.84 ± 9.48	48.96 ± 11.86	44.41 ± 10.80	50.72 ± 11.61
Trapezius	36.38 ± 10.50	35.78 ± 11.26	32.85 ± 12.83	26.85 ± 12.75
Leg-flipping phase				
Tibialis anterior	125.37 ± 26.33	141.82 ± 23.38	132.74 ± 28.96	140.23 ± 30.74
Gastrocnemius medialis	60.74 ± 11.87	62.61 ± 11.35	58.95 ± 10.89	54.85 ± 9.73
Gastrocnemius lateralis	58.43 ± 10.95	64.82 ± 13.90	59.70 ± 11.51	63.72 ± 12.87
Adductor longus	88.09 ± 13.42	103.11 ± 10.81	85.72 ± 12.84	97.31 ± 13.74
Adductor magnus	92.48 ± 18.96	121.65 ± 20.98 *	94.86 ± 17.07	115.69 ± 21.62
Biceps femoris	54.26 ± 14.62	63.86 ± 17.02	55.27 ± 14.89	67.77 ± 19.83
Gluteus maximus	60.63 ± 12.87	61.72 ± 14.63	65.86 ± 16.67	69.74 ± 18.82
Rectus abdominis	136.38 ± 37.48	157.38 ± 38.26 *	130.21 ± 42.96	143.68 ± 40.21
Latissimus dorsi	80.59 ± 26.24	86.69 ± 25.17	79.74 ± 31.92	77.85 ± 27.95
Trapezius	75.44 ± 27.48	81.66 ± 23.57	77.87 ± 25.26	80.84 ± 27.03
Pedal-clamp stage				
Tibialis anterior	132.65 ± 30.69	116.15 ± 35.96 *	133.62 ± 37.89	125.82 ± 41.85
Gastrocnemius medialis	95.36 ± 31.35	101.74 ± 25.63	91.71 ± 30.80	99.38 ± 27.63
Gastrocnemius lateralis	104.47 ± 30.59	127.37 ± 27.69 *	112.85 ± 33.84	123.76 ± 30.09
Adductor longus	87.68 ± 26.48	93.46 ± 23.65	81.69 ± 22.55	88.72 ± 20.18
Adductor magnus	80.30 ± 28.59	78.85 ± 20.12	83.45 ± 29.95	84.79 ± 23.72
Biceps femoris	49.48 ± 19.06	52.66 ± 20.88	51.76 ± 22.38	49.79 ± 19.04
Gluteus maximus	92.94 ± 23.48	96.41 ± 23.67	90.12 ± 27.67	98.36 ± 26.15
Rectus abdominis	55.08 ± 17.21	57.87 ± 16.06	51.03 ± 15.88	60.36 ± 16.86
Latissimus dorsi	53.40 ± 16.08	49.88 ± 19.62	46.21 ± 17.87	49.07 ± 20.39
Trapezius	40.57 ± 17.29	43.38 ± 20.17	44.46 ± 18.72	40.17 ± 19.83
Gliding phase				
Tibialis anterior	38.42 ± 9.02	33.11 ± 8.57	35.74 ± 10.11	34.20 ± 9.08
Gastrocnemius medialis	20.52 ± 9.50	19.76 ± 10.26	22.80 ± 9.43	25.86 ± 10.36
Gastrocnemius lateralis	19.53 ± 8.43	20.63 ± 8.96	19.72 ± 9.57	19.36 ± 10.48
Adductor longus	20.48 ± 9.92	24.67 ± 8.92	19.37 ± 10.10	20.56 ± 9.88
Adductor magnus	21.48 ± 10.49	17.59 ± 11.69	20.77 ± 9.04	19.50 ± 9.63
Biceps femoris	29.53 ± 6.02	24.72 ± 10.59	27.69 ± 7.36	28.63 ± 8.79
Gluteus maximus	20.92 ± 9.39	24.72 ± 10.73	21.73 ± 8.50	22.63 ± 9.61
Rectus abdominis	46.35 ± 11.70	44.80 ± 12.63	47.18 ± 12.34	46.01 ± 11.26
Latissimus dorsi	33.33 ± 10.74	34.72 ± 12.05	34.84 ± 10.17	33.92 ± 11.90
Trapezius	29.38 ± 10.80	30.00 ± 11.09	26.57 ± 12.29	28.79 ± 10.92

Note: *: Time × Group interaction effect, *p* < 0.05.

**Table 5:** Muscle Synergy Structures for Synergy Pattern SYN1 in Breaststroke Movements.

SYN1%	Experimental Group		Control Group	
	Pre-Intervention	Post-Intervention	Pre-Intervention	Post-Intervention
Tibialis anterior	0.08 ± 0.04	0.11 ± 0.05	0.04 ± 0.08	0.07 ± 0.05
Gastrocnemius medialis	0.77 ± 0.17	0.75 ± 0.20	0.84 ± 0.21	0.87 ± 0.14
Gastrocnemius lateralis	0.79 ± 0.12	0.82 ± 0.18	0.86 ± 0.06	0.90 ± 0.17
Adductor longus	0.31 ± 0.07	0.40 ± 0.12	0.35 ± 0.17	0.41 ± 0.10
Adductor magnus	0.44 ± 0.13	0.52 ± 0.15	0.40 ± 0.10	0.45 ± 0.14
Biceps femoris	0.09 ± 0.03	0.07 ± 0.03	0.11 ± 0.08	0.14 ± 0.05
Gluteus maximus	0.55 ± 0.24	0.80 ± 0.07	0.49 ± 0.27	0.58 ± 0.15
Rectus abdominis	0.30 ± 0.16	0.35 ± 0.14	0.33 ± 0.29	0.31 ± 0.13
latissimus dorsi	0.20 ± 0.10	0.16 ± 0.07	0.16 ± 0.23	0.14 ± 0.10
trapezius	0.13 ± 0.19	0.19 ± 0.09	0.16 ± 0.21	0.20 ± 0.16

**Table 6:** Muscle Synergy Patterns for SYN2 in the Breaststroke Movement.

SYN2%	Experimental Group		Control Group	
	Pre-Intervention	Post-Intervention	Pre-Intervention	Post-Intervention
Tibialis anterior	0.89 ± 0.16	0.71 ± 0.07	0.93 ± 0.18	0.83 ± 0.14
Gastrocnemius medialis	0.11 ± 0.05	0.14 ± 0.08	0.16 ± 0.03	0.09 ± 0.02
Gastrocnemius lateralis	0.22 ± 0.14	0.20 ± 0.09	0.25 ± 0.11	0.23 ± 0.06
Adductor longus	0.26 ± 0.11	0.29 ± 0.03	0.29 ± 0.13	0.25 ± 0.10
Adductor magnus	0.27 ± 0.08	0.26 ± 0.11	0.30 ± 0.11	0.24 ± 0.12
Biceps femoris	0.44 ± 0.25	0.48 ± 0.20	0.50 ± 0.31	0.48 ± 0.26
Gluteus maximus	0.26 ± 0.18	0.24 ± 0.13	0.22 ± 0.24	0.16 ± 0.13
Rectus abdominis	0.25 ± 0.16	0.27 ± 0.10	0.29 ± 0.18	0.25 ± 0.17
latissimus dorsi	0.21 ± 0.13	0.25 ± 0.17	0.17 ± 0.14	0.23 ± 0.11
trapezius	0.24 ± 0.10	0.20 ± 0.07	0.21 ± 0.17	0.24 ± 0.05

**Table 7:** Muscle Synergy Structures for Synergy Pattern SYN3 in Breaststroke Movements.

SYN3%	Experimental Group		Control Group	
	Pre-Intervention	Post-Intervention	Pre-Intervention	Post-Intervention
Tibialis anterior	0.13 ± 0.05	0.10 ± 0.07	0.09 ± 0.08	0.06 ± 0.06
Gastrocnemius medialis	0.27 ± 0.15	0.21 ± 0.09	0.21 ± 0.19	0.18 ± 0.07
Gastrocnemius lateralis	0.22 ± 0.18	0.16 ± 0.12	0.14 ± 0.17	0.22 ± 0.13
Adductor longus	0.15 ± 0.10	0.16 ± 0.08	0.18 ± 0.15	0.24 ± 0.10
Adductor magnus	0.22 ± 0.17	0.27 ± 0.13	0.26 ± 0.20	0.30 ± 0.16
Biceps femoris	0.10 ± 0.09	0.15 ± 0.10	0.12 ± 0.14	0.10 ± 0.11
Gluteus maximus	0.16 ± 0.13	0.22 ± 0.16	0.14 ± 0.15	0.17 ± 0.10
Rectus abdominis	0.62 ± 0.10	0.93 ± 0.06	0.76 ± 0.13	0.78 ± 0.15
latissimus dorsi	0.74 ± 0.17	0.79 ± 0.08	0.85 ± 0.22	0.84 ± 0.18
trapezius	0.65 ± 0.27	0.70 ± 0.21	0.62 ± 0.28	0.57 ± 0.25

**Table 8:** Muscle Synergy Patterns in the SYN4 Synergy Mode During Breaststroke Movement.

SYN4%	Experimental Group		Control Group	
	Pre-Intervention	Post-Intervention	Pre-Intervention	Post-Intervention
Tibialis anterior	0.05 ± 0.03	0.11 ± 0.07	0.02 ± 0.05	0.06 ± 0.04
Gastrocnemius medialis	0.60 ± 0.27	0.71 ± 0.24	0.52 ± 0.31	0.46 ± 0.26
Gastrocnemius lateralis	0.54 ± 0.23	0.49 ± 0.20	0.57 ± 0.27	0.59 ± 0.25
Adductor longus	0.68 ± 0.17	0.92 ± 0.08	0.76 ± 0.25	0.84 ± 0.19
Adductor magnus	0.75 ± 0.15	0.88 ± 0.16	0.78 ± 0.20	0.83 ± 0.15
Biceps femoris	0.17 ± 0.13	0.41 ± 0.15	0.23 ± 0.17	0.32 ± 0.15
Gluteus maximus	0.26 ± 0.22	0.37 ± 0.20	0.30 ± 0.21	0.33 ± 0.17
Rectus abdominis	0.15 ± 0.10	0.23 ± 0.13	0.12 ± 0.11	0.08 ± 0.07
latissimus dorsi	0.16 ± 0.07	0.10 ± 0.05	0.10 ± 0.13	0.15 ± 0.14
trapezius	0.20 ± 0.14	0.27 ± 0.17	0.15 ± 0.10	0.18 ± 0.11

**Table 9:** Activation Coefficient of Synergistic Pattern SYN1 in Breaststroke Movement.

Activation Coefficient	Parameters	Experimental Group		Control Group	
		Pre-Intervention	Post-Intervention	Pre-Intervention	Post-Intervention
SYN1	Activation Duration T	0.44 ± 0.15	0.35 ± 0.09	0.37 ± 0.11	0.31 ± 0.13
	Peak Moment Tmax	0.30 ± 0.09	0.32 ± 0.14	0.27 ± 0.10	0.25 ± 0.08
	The Moment Begins Tstart	0.11 ± 0.07	0.15 ± 0.05	0.08 ± 0.04	0.14 ± 0.06
SYN2	Activation Duration T	0.65 ± 0.27	0.34 ± 0.15	0.61 ± 0.33	0.50 ± 0.26
	Peak Moment Tmax	0.36 ± 0.23	0.49 ± 0.27	0.39 ± 0.21	0.40 ± 0.17
	The Moment Begins Tstart	0.18 ± 0.16	0.38 ± 0.10	0.21 ± 0.15	0.27 ± 0.12
SYN3	Activation Duration T	0.50 ± 0.17	0.47 ± 0.13	0.54 ± 0.22	0.51 ± 0.20
	Peak Moment Tmax	0.37 ± 0.12	0.45 ± 0.16	0.44 ± 0.16	0.46 ± 0.14
	The Moment Begins Tstart	0.18 ± 0.08	0.10 ± 0.14	0.24 ± 0.10	0.20 ± 0.11
SYN4	Activation Duration T	0.63 ± 0.25	0.60 ± 0.17	0.68 ± 0.30	0.62 ± 0.21
	Peak Moment Tmax	0.75 ± 0.28	0.71 ± 0.26	0.70 ± 0.35	0.64 ± 0.28
	The Moment Begins Tstart	0.39 ± 0.16	0.45 ± 0.20	0.32 ± 0.15	0.37 ± 0.20

## Data Availability

The original contributions presented in this study are included in the article. Further inquiries can be directed to the corresponding author.
